# Synthesis and Characterization of Mithun (*Bos frontalis*) Urine-Based Antibacterial Copper Oxide Nanoparticles

**DOI:** 10.3390/biomedicines11061690

**Published:** 2023-06-11

**Authors:** Khriebu Bizo Pelesinuo, Govindharajan Sattanathan, Nazrul Haque, Khalid A. Al-Ghanim, Marcello Nicoletti, Nadezhda Sachivkina, Marimuthu Govindarajan

**Affiliations:** 1 Department of Zoology, St. Joseph University, Chumoukedima 797115, Nagaland, India; pelesinomeyase@gmail.com (K.B.P.); sattanathanphd@gmail.com (G.S.); 2 ICAR—National Research Centre on Mithun, Medziphema 797106, Nagaland, India; nazrulhaq63@gmail.com; 3 Department of Zoology, College of Science, King Saud University, Riyadh 11451, Saudi Arabia; kghanim@ksu.edu.sa; 4 Department of Environmental Biology, Sapienza University of Rome, 00185 Rome, Italy; marcello.nicoletti@uniroma1.it; 5 Department of Microbiology V.S. Kiktenko, Institute of Medicine, Peoples Friendship University of Russia Named after Patrice Lumumba (RUDN University), 117198 Moscow, Russia; 6 Unit of Mycology and Parasitology, Department of Zoology, Annamalai University, Annamalainagar 608002, Tamil Nadu, India; 7 Unit of Natural Products and Nanotechnology, Department of Zoology, Government College for Women (Autonomous), Kumbakonam 612001, Tamil Nadu, India

**Keywords:** nanotechnology, biosynthesis, cow urine, biomedicine, *Aeromonas hydrophila*, *Aeromonas veronii*

## Abstract

The increased prevalence of disease, mortality, and antibiotic resistance among aquatic microorganisms has renewed interest in non-conventional disease prevention and control approaches. Nanoparticles present several benefits in aquaculture and hold significant potential for controlling both human and animal infections. This study reports on the antibacterial properties of green copper oxide nanoparticles (CuO NPs) synthesized from the urine of Mithun (MU) (*Bos frontalis*). In addition, an array of analytical techniques, including scanning electron microscopy (SEM), X-ray diffraction (XRD), UV-visible spectroscopy (UV), and Fourier transform infrared spectroscopy (FTIR), were employed to investigate the synthesized MU-CuO nanoparticles. *Aeromonas hydrophila* and *Aeromonas veronii*, two bacterial fish pathogens known to cause severe infectious diseases in fish, were tested for their antibacterial efficacy against MU-CuO NPs. At 100 µg/mL, MU-CuO NPs exhibit enhanced antibacterial efficacy against two bacterial pathogens commonly found in fish. Applications in aquaculture may be looked at given that MU-CuO NPs showed greater antibacterial activity.

## 1. Introduction

Fish are prone to various illnesses, and the primary cause of fish mortality is attributed to diseases, particularly in juvenile fish. Fish diseases can be classified into two categories, namely pathogenic and non-pathogenic, based on their level of contagiousness. The ultimate state, which is associated with inadequate water quality, malnourishment, and other related factors, is non-communicable in fish. Non-communicable diseases encompass a range of conditions that may arise due to factors such as hyper aeration, inadequate nutrient intake (including vitamin and mineral deficiencies), exposure to industrial and agricultural pollutants, genetic mutations, and neoplastic anomalies, which involve abnormal growth and development of organs resulting in their structural and functional impairment [1,2,3,4]. The alternative form of ailment is pathogenic in nature and is believed to be especially deleterious due to its propensity for transmission between fish, ultimately leading to substantial mortality rates.

The utilization of chemotherapeutic and antimicrobial agents has been deemed a critical approach in preventing and managing infectious pathogens, with considerable attention devoted to this area in recent decades [5]. Nonetheless, the frequent utilization of these medications presents a range of disadvantages. Aquatic fauna is also susceptible to the significant adverse impacts of antibiotic resistance on all living organisms [6]. The scientific community has extensively substantiated the potential threat to human well-being that arises from the unselective application of antibiotics in aquatic organisms such as fish. When antibiotics are used too much, they hurt the environment and living processes [7,8]. Moreover, it often facilitates the emergence and dissemination of infections that exhibit elevated levels of drug resistance. This increases the probability of horizontal gene transfer [9,10]. To effectively manage infections, it is imperative to explore alternative strategies.

Metal nanoparticles have several potential applications in nanomedicine and biotechnology. In addition to being effective against viruses and inflammation, it also inhibits the growth of biofilms and bacteria and promotes the healing of wounds [11]. The aforementioned phenomenon provides a substantial surface area for the management of biochemical reactions occurring at various levels within pathogenic cells and simultaneously influences multiple alterations in bacterial metabolic mechanisms [12]. The amalgamation of metallic nanoparticles and antibiotics can potentially augment the antimicrobial properties of both constituents through synergistic and reinforced performance, as suggested by previous research [13]. Metal oxide nanoparticles, including Al_2_O_3_, ZnO, MgO, CuO, TiO_2_, and CaO, have been synthesized and are recognized for their intrinsic antimicrobial properties [14]. Inorganic oxide nanoparticles offer sKarthika everal advantages over organic antimicrobial agents, including enhanced stability, robustness, and superior efficacy, against resistant microbial pathogens [15,16,17]. Copper oxide nanoparticles (CuO NPs) have unique characteristics and several biomedical uses [18,19]. Several methods have been employed to synthesize CuO NPs [20,21,22,23]. The relevance of CuO NPs has significantly increased due to their simplicity and unique capabilities in the fields of optics, electricity, and pharmacology [24]. The utilisation of CuO NPs has been observed to demonstrate functional properties such as antibacterial, antibiotic, antifungal, and antifouling agents in various biological and biomedical applications [25].

Cows are regarded in Indian tradition as sacred and venerable creatures known as “KAMADHENU”, which translates to “the mother of all spiritual entities”, from the dawn of time. In India, panchagavya, which comprises of cow urine, ghee, milk, curd, and dung, is utilised as a dietary supplement, spiritual aid, and medicine owing to its reputed legendary, spiritual, and medical significance [26,27]. According to recent research [28], cow urine has been suggested as a potential remedy for various health conditions, including joint pain, high blood pressure, diabetes, heart disease, cancer, thyroid disorders, asthma, psoriasis, skin inflammation, headache, ulcer, and gynaecological issues. According to ancient Ayurvedic literature, cow urine contains 95% water, 2.5% urea, and 2.5% minerals, salts, hormones, and enzymes [26]. Ancient Indian Ayurvedic literature suggests using cows’ liquid metabolic waste to treat chronic diseases. Cow urine is anti-neoplastic, according to medical authorities [27]. Additionally, cow urine exhibits promising medicinal properties in the fields of antimicrobial, antioxidant, anti-anthelmintic, anticancer, and biosensors, which are significant in the realm of biotechnology. According to research findings, cow urine has been established as a potent and effective therapeutic agent capable of treating diverse medical conditions [28].

Cow urine (CU) is a component of panchagavya, an ancient Ayurvedic system in India, due to its purported therapeutic properties. The potential to act as a reducing agent is given to CU by the existence of different biomolecular entities within it [14]. The present research introduces a new approach to employing copper nanoparticles by means of incorporating MU integration. The utilization of the synthesis strategy is distinguished by its uncomplicated nature, economic viability, and ecological sustainability. The biomolecules within MU serve as capping agents, thereby playing a role in the stabilization of the nanoparticles. The present investigation examined the antibacterial properties of MU-CuO NPs derived from Mithun urine against *Aeromonas hydrophila* and *Aeromonas veronii.*

## 2. Materials and Methods

### 2.1. Chemicals

Copper sulphate (CuSO_4_·5H_2_O) of analytical quality was purchased from Merck in Electronic City, India. All of the procedures in the experiment were carried out using double-distilled (DD) water.

### 2.2. Collection of Mithun Urine

The Mithun urine sample was obtained from the Mithun breeding centre, ICAR—National Research on Mithun, located in Medziphema, Nagaland, India. Following the filtration of the Mithun urine sample using Whatman filter paper No. 1, the resulting filtrate was stored under refrigeration until its utilization in subsequent analyses.

### 2.3. Preparation of MU-CuO NPs

A 10 mM solution of copper sulphate, comprising 50 mL, was introduced to 5 mL of freshly filtered urine from Mithun in a 100 mL Erlenmeyer flask. The mixture was subjected to continuous stirring at a temperature range of 100–120 °C, facilitated by a magnetic stirrer. The colour of the reaction mixture underwent several changes, transitioning from a deep blue hue to colourless, followed by a shift from colourless to brick red, and ultimately culminating in a dark red shade after undergoing vigorous stirring for a duration of 24 h. Subsequently, the resultant amalgamation was subjected to centrifugation for a duration of 10 min at ambient temperature and a speed of 10,000 revolutions per minute, utilizing a Beckman centrifuge equipped with a Beckman JA-17 rotor. The supernatant was eliminated prior to the collection of the amalgamated substance. The MU-CuO NPs were air-dried on a watch glass after collection. The resulting black precipitate was subjected to grinding in order to enhance its distinctive properties.

### 2.4. Microorganisms

The bacterial strains used in the research were *A. veronii* (MTCC 3249) and *A. hydrophila* (MTCC 646). We obtained both bacterial strains from IMTEH in Chandigarh, India. 

### 2.5. Characterization of Synthesized MU-CuO NPs 

The production of MU-CuO NPs was monitored by UV-visible spectroscopy (Make, Perkin Elmer, Waltham, MA, USA). An FTIR spectrophotometer (Perkin Elmer Model RXI) and the KBr pellet technique were used to verify the presence of functional groups. The shape and elemental content of synthesized CuO NPs were studied using scanning electron microscopy (SEM) Model FEG-Quanta 250 with energy dispersive atomic X-ray spectroscopy (EDAX). The XRD patterns were captured using a Philips analytical X-ray diffractometer with a CuK radiation (λ = 0.15418) source at 40 KV and 30 MA.

### 2.6. Antibacterial Activity

Gram-negative fish pathogenic strains, including *A. hydrophila* (MTCC 646) and *A. veronii* (MTCC 3249), were investigated for antibacterial activity using an agar well diffusion technique with green-generated MU-CuO NPs. The MIC of the MU-CuO NPs against each bacterial pathogen was determined using the micro-dilution broth technique. All bacterial dilutions were standardized using the McFarland (turbidity) standard, and the resultant bacterial density was 1.5 × 10^8^ CFU/mL. The agar well diffusion technique [28] was followed by Cappuccino and Sherman [29] to examine the antibacterial activity. Different quantities of MU-CuO NPs (T1-25, T2-50, T3-75, and T4-100 µg/mL; positive control as Gentamycin; negative control as DMSO) were injected in the wells of cultivated agar plates using a sterile well puncture. After adding MU-CuO NPs to the culture plates, the plates were placed in a 37 °C incubator for 24 h. Following incubation, the plates were examined for the presence of zones, indicative of the nanoparticles’ inhibitory effect on the bacterial pathogens. The areas were measured using a millimeter ruler, and the results were compared.

### 2.7. Statistical Analysis

The data were subjected to one-way analysis of variance (ANOVA) for statistical analysis. The concentrations were subjected to multiple comparisons using Duncan’s multiple-range tests to assess any variations. The statistical analyses were conducted utilizing SPSS software (Version 21). The statistical significance threshold was established at a level of *p* < 0.05, and the outcomes were presented as the mean value accompanied by the standard error.

## 3. Results

### 3.1. UV-Visible Spectroscopic Analysis 

**Figure 1:**
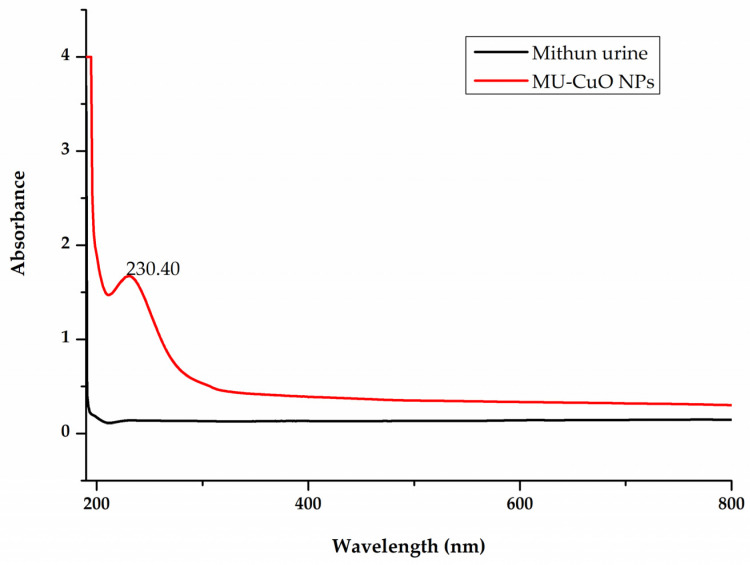
The UV-visible spectra of green synthesized MU-CuO NPs.

The UV-visible spectrum of the MU-CuO NPs that were synthesized is illustrated in Figure 1. The UV-visible spectral analysis provides evidence for the formation and endurance of MU-CuO NPs in a colloidal solution in an aqueous environment. The plots depicted the relationship between absorbance and wavelength. The wavelength of 230.40 nm exhibits a notable increase in the absorption spectra of MU-CuO nanoparticles.

### 3.2. Fourier Transform Infrared Spectroscopic Analysis

**Figure 2:**
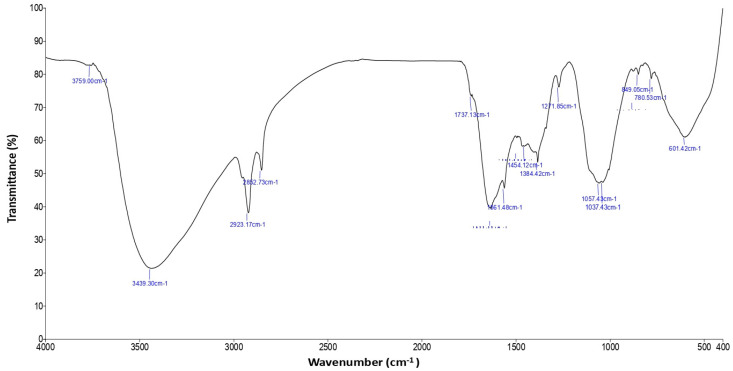
The FTIR spectra of synthesized MU-CuO NPs.

The FTIR spectrum of MU-CuO NPs is illustrated in Figure 2. The study employed FTIR analysis to examine the presence of functional groups in MU-CuO NPs. The broad absorption peak observed at approximately 2923.17 cm^−1^ can be attributed to the adsorbed water molecules. 3759.00 cm^−1^ is the phenolic compound’s -OH group stretching vibrations. The stretching modes of -CH_2_ and C-H in alkanes were assigned to the spectral bands at 1737.13 cm^−1^ and 1454.12 cm^−1^, respectively.

### 3.3. Scanning Electron Microscopic Analysis

**Figure 3:**
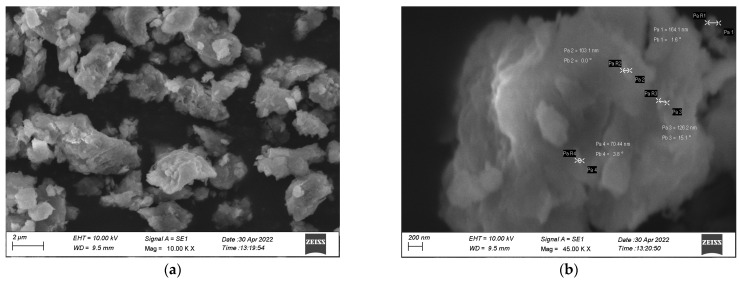

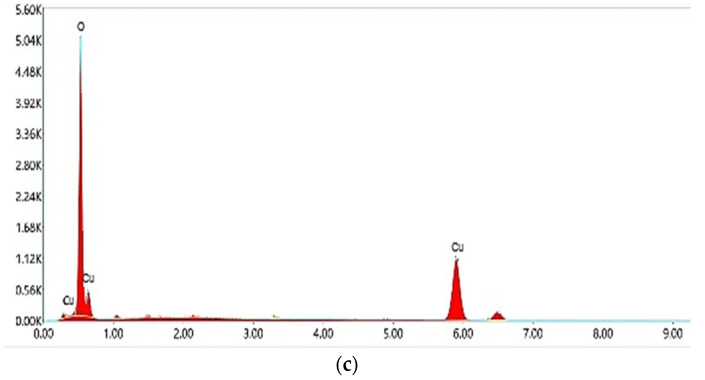
The SEM (**a**,**b**) and EDX (**c**) micrographs show the surface morphology of synthesized MU-CuO NPs.

Figure 3a,b, illustrates the examination of the synthesized MU-CuO NPs through the utilization of scanning electron microscopy utilizing Mithun urine. The MU-CuO NPs were agglomerated with a particle size in the range of 44–56 nm. Various forms of MU-CuO NPs were synthesized due to bioactive constituents in Mithun urine. The elemental composition of MU-CuO NPs is seen in the EDAX spectra shown in Figure 3c.

### 3.4. X-ray Diffraction Analysis

**Figure 4:**
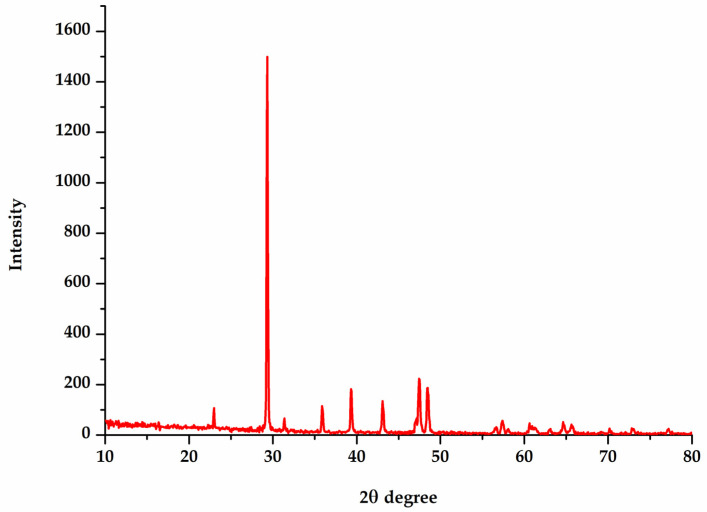
The XRD analysis of MU-CuO NPs.

The XRD technique was employed to confirm the crystalline structure of the MU-CuO NPs (Figure 4). The reflection at 2θ values 29.32, 35.89, 39.33, 43.11, 47.48, and 48.47, which are indexed to be lattice planes with (110), (111), (200), (220), (301), and (204) individually, was proven by the XRD pattern of synthesized MU-CuO NPs. The average size of the crystals was determined from the XRD data, utilizing the Scherrer equation (D = 0.94 × λ/β × Cosθ). The mean size of the crystals was determined to be 48.47.

### 3.5. Antibacterial Analysis

**Figure 5:**
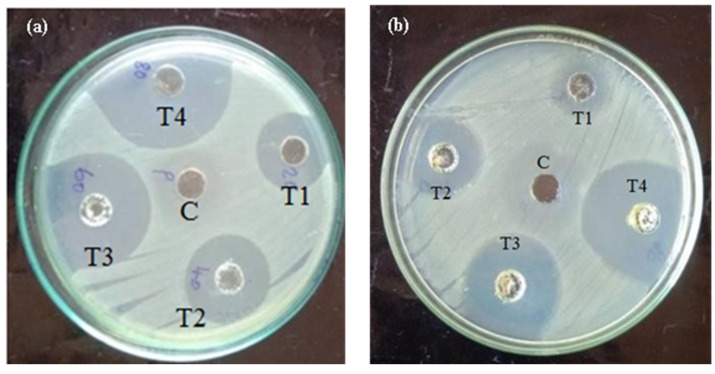
Antibacterial activity of Mithun urine CuO NPs against (**a**) *Aeromonas hydrophila* and (**b**) *A. veronii* (different concentrations of MU-CuO NPs T1-25, T2-50, T3-75, and T4-100 µg/ mL; positive control as Gentamycin).

**Figure 6:**
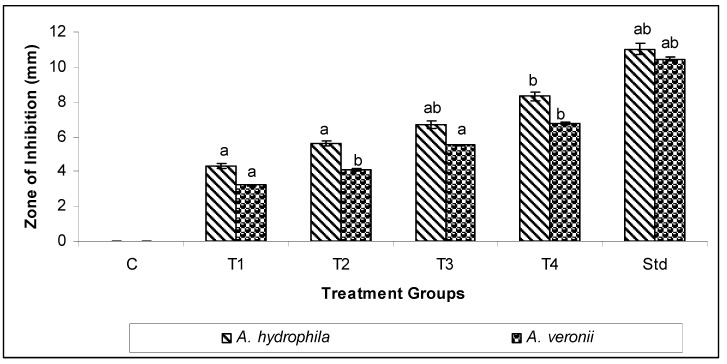
Antibacterial activity of Mithun urine-based CuO NPs against selected fish bacterial pathogens (different concentrations of MU-CuO NPs T1-25, T2-50, T3-75, and T4-100 µg/mL; C-DMSO; Std—Gentamycin). Differences between experimental groups that are statistically significant (*p* ≤ 0.05) correspond to distinct alphabet letters.

Two fish pathogenic bacteria, shown in Figure 5 and Figure 6, were used to test the antibacterial activity of MU-CuO NPs. The study observed that the highest level of zone inhibition was recorded in T4 at 8.32 ± 0.12 mm, while *A. hydrophila* and *A. veronii* exhibited zone inhibitions of 6.74 ± 0.09 mm and T4, respectively. The results of our study indicate that MU-CuO NPs exhibit superior antibacterial efficacy.

## 4. Discussion

Nanotechnology is a burgeoning field that has many potential uses in fields as diverse as agriculture, food production, environmental protection, and healthcare administration [30,31,32]. Nanoparticles have demonstrated remarkable efficacy in all of their socially advantageous applications over the course of their existence. Nanoparticles’ exceptional qualities make them superior to their bigger counterparts in a number of ways [33]. Nanoparticles composed of metals and/or their oxides have garnered attention as potential antibacterial agents due to their physicochemical properties. These nanoparticles can eradicate infections in various ways, such as by inhibiting normal cellular functions or preventing the synthesis of helpful macromolecules [34].

Fish exhibit susceptibility to a diverse array of microbial agents, with bacterial infections being the predominant etiological factor and a major contributor to substantial economic losses worldwide. Fungal infections are commonly observed in circumstances of heightened stress and in regions characterized by suboptimal water quality [35]. Furthermore, cyanobacterial blooms have been observed to cause significant harm to aquaculture on a global scale, with microcystins being particularly detrimental to fish at concentrations as low as a few micrograms [36,37,38,39]. In addition, as an additional way, the antibacterial activity of a broad range of nanoparticles that are both commercially available and synthesized in the laboratory was evaluated and compared to the efficacy of a selection of hazardous pathogens [40,41]. The investigators in this study synthesized the CuO NPs in a lab. The research found that the most significantly synthesized CuO NPs had a mean particle percentage of 128.25% and a peak size of 230.33 nm [42]. 

The nanoparticles that were synthesized exhibited antibacterial properties and have the potential to enhance antimicrobial agents. Various types of nanoparticles, including silver, zinc, magnesium, and calcium nanoparticles, have been investigated for their potential applications as agents for cancer treatment [43,44,45] and wound healing [46]. The copper oxide nanoparticles (CuO NPs) derived from Mithun urine exhibit several properties that render them potentially useful in various fields. The current study has demonstrated that MU-CuO NPs exhibit antibacterial properties. MU-CuO NPs are greenly synthesized by mixing an aqueous solution of copper sulphate with MUD and incubating for 10 h at room temperature. CuO NPs produced when Cu ions are reduced, resulting in a deeper blue colour. UV-visible spectroscopy identified MU-CuO NPs. The UV-visible spectrum had a large peak at 280 nm, boosting MU-CuO NP synthesis [47]. The reaction mixture’s UV-visible peak did not rise after 10 h incubation, demonstrating its completion (Figure 1).

The FTIR analysis of bovine urine revealed a conspicuous peak at 3439.30, 2923.17, 2852.73, 1737.13, and 1384.42 cm^−1^, along with highly broadened peaks around 3759.00 cm^−1^, indicating the stretching of -O-H bonds that correspond to polyhydroxy compounds such as lactose and cresol. A prominent and wide peak was observed at 2923.17 cm^−1^, indicating the -O-H stretching vibration characteristic of a carboxylic acid. The spectral analysis reveals a distinct and concentrated band at approximately 1737.13 cm^−1^, which corresponds to the stretching of the carbonyl group of amide (-CO-NH). Additionally, a sharp peak at 1510.02 cm^−1^ is observed, which indicates the presence of a benzene ring in an aromatic compound. The spectral peak located at a wavenumber of 1336.32 cm^−1^ corresponds to the stretching vibration of the carboxylic group’s carbonyl functional group. The FTIR analysis of MU-CuO NPs revealed significant peaks at 1661.48, 1271.85, and 1037.43 cm^−1^. Additionally, a band was observed at 849.05 cm^−1^, indicating the stretching vibration of the -N-H group, which is consistent with the presence of amides (Figure 2).

The scanning electron microscopy (SEM) image depicted in Figure 3a illustrates that most of the MU-CuO NPs exhibit agglomerated spherical morphology and tend to aggregate, forming clusters and showing a size range of 44–56 nm. The MU-CuO NPs were subjected to X-ray diffraction (XRD) analysis after undergoing shade drying at ambient temperature. The XRD pattern of the synthesized MU-CuO NPs exhibited reflections at 2θ values of 2θ values 29.32, 35.89, 39.33, 43.11, 47.48, and 48.47, which are indexed to be lattice planes with (110), (111), (200), (220), (301), and (204), respectively. The average size of the crystals was determined from the XRD data, utilizing the Scherrer equation. The mean size of the crystals was determined to be 48.47. These values are consistent with those reported in the literature [48]. The broadening of the peak observed in MU-CuO NPs indicates the presence of small nanocrystals in the samples. The wide peak also shows that the specimen lacks bulk materials. Figure 4 shows impurity-induced peaks. 

The investigation into the antibacterial efficacy of MU-CuO NPs against *A. hydrophila* and *A. veronii* through disc diffusion tests revealed the antimicrobial properties of MU-CuO NPs. The outcomes of the disc diffusion experiments elucidated distinct zones of inhibition (ZOI) for varying concentrations of MU-CuO NPs, indicating a robust correlation between the dose and ZOI (refer to Figure 5a,b). The results suggest that the concentration of 100 μg/μL of MU-CuO NPs exhibited the most significant antibacterial activity, as evidenced by the observed zone inhibition. Specifically, the highest zone inhibition was recorded for T4 (8.32 ± 0.12 mm) against *A. hydrophila*, followed by T4 (6.74 ± 0.09 mm) against *A. veronii*.

The present investigation demonstrated MU CuO nanoparticles’ bactericidal efficacy against all the assessed bacterial agents. Prior research has indicated that CuO nanoparticles exhibit similar antibacterial efficacy against diverse bacterial strains [49,50,51]. The CuO NPs showed a size of 40 nm, whereas the lab-manufactured CuO NPs had a larger size of B93 nm. The latter demonstrated greater antibacterial efficacy and lower minimum inhibitory concentration (MIC) values compared to commercial CuO NPs. The findings of the present investigation diverge from those of Sohail et al. [52], as their research revealed a correlation between the dimensions of CuO NPs and their antimicrobial efficacy. Additionally, the researchers discovered that utilizing CuO NPs, having a size of either 20 or 1.24 nanometers, resulted in a noteworthy decrease in the incidence of infections. The aggregation of CuO NPs in an aqueous environment of commercial nature may impede the direct interaction between the particles and microorganisms. The various dissolving properties may account for this variation in antibacterial effectiveness [53,54,55].

Nanomaterials that are mediated by cow urine exhibit a wide range of properties that render them suitable for deployment in diverse applications. As a result, we have emphasized their noteworthy results and guided for future researchers to underscore their beneficial applications in anti-asthmatic, antibacterial, antioxidant, anticancer, and photocatalytic activities. In a study conducted by Sumathy and Babujanarthanam [56], cow urine nanoparticles (NPs) were synthesized with acebrophylline. The generated NPs had an overall agglomerated spherical shape. The anti-asthmatic properties of said nanoparticles were subsequently examined. Meghnath Prabhu et al. [57] used aged cow urine to reduce Ag NPs in a one-pot green synthesis. Synthesized nanoparticles were tested for antibacterial characteristics. Sonication at pH 9.5 for one minute produced Ag NPs quickly. 

Jain and colleagues [58] conducted an investigation into the hydrothermal synthesis of silver nanoparticles (AgNPs) utilizing cow urine and honey as reducing agents and subsequently assessed their antibacterial efficacy. *Pseudomonas* sp. bacteria were stopped in their tracks by AgNPs that were artificially produced. Panchagavya and silver nitrate were used in the manufacture of spherical AgNPs by Govarthanan and colleagues [59]. The formation of AgNPs was confirmed by XRD, SEM, and TEM. Nanoparticles were synthesized, and tests showed that they were efficient against *Aeromonas*, *Acinetobacter*, and *Citrobacter* sp.

Arumugam et al. [60] revealed that Panchagavya-mediated Cu NPs production was inexpensive, simple, ecologically friendly, and non-toxic. Cu NPs were tested for brine shrimp cytotoxicity. Vinay et al. [61] studied the antibacterial and catalytic properties of AgNPs and presented a straightforward technique for their synthesis using cow urine without hazardous chemicals. Synthesized AgNPs exhibited potential antibacterial properties. Nazeruddin et al. [62] conducted a concentration-dependent study on dangerous bacteria utilising cow urine to make CdNPs. The work by Suk et al. [63] outlined the microemulsion procedure for encapsulating cellulose nanoparticles in cow urine and investigated their antibacterial effectiveness against pathogenic bacteria. 

Satheeshkumar et al. [64] synthesized spherical CuFe_2_O_4_ NPs using sol-gel and cow urine as a chelating agent. 14.5–22.3 nm CuFe_2_O_4_ NPs. Chamoli et al. made GNs from urea and cow urine [65]. Synthetic GNs performed better electrically and optically. Prasad et al. [66] bio-inspired Pd NPs from cow urine. The produced NPs were tested for antibacterial, antioxidant, and catalytic properties. 

This work discusses the creation of copper oxide nanoparticles from Mithun urine as well as their antibacterial effectiveness against gram-negative microorganisms. The synthetic methodology is characterized by its simplicity, affordability, and cost-efficiency. The outcomes of the ecologically sustainable approach for synthesizing CuO NPs exhibit promising biological properties. Consequently, the CuO NPs produced in an environmentally friendly manner exhibit potential utility in the realm of biomedicine. This study focused on the eco-friendly production of CuO NPs and examined their efficacy as an antibacterial agent against bacterial pathogens in fish. Mithun Urine as a precursor in the synthesis process yields nanoparticles with precise control over their size and morphology. XRD and SEM studies confirmed the formation of nano CuO with a cluster shape and a crystallite size of 70.44 nm. The antibacterial properties were evaluated and demonstrated significant efficacy against bacterial pathogens affecting fish. The characteristics mentioned above of CuO NPs render them a promising antibacterial agent with potential applications in aquaculture. Finally, the present research has shown that nanoparticles, in particular, produced CuO, have exceptional antibacterial effects and may be further examined as an alternative to antibiotics in aquaculture. It is critical to learn how metal nanoparticles inhibit the growth of microorganisms used in aquaculture. Today’s studies concentrate on developing environmentally friendly and economically viable methods of manufacturing nanomaterials. Aquaculture and other environmental and health issues highlight the need for laboratory research to be scaled up to industrial levels. Natural, non-hazardous nanomaterials, like Mithun urine, are the current focus of nanotechnology research. This environmentally friendly strategy might be effective in practise.

## 5. Conclusions

The feasibility of employing copper oxide nanoparticles sourced from Mithun urine as an effective antibacterial agent to counteract bacterial pathogens in fish has been proposed. The MU-CuO nanoparticles exhibited notable bactericidal efficacy at a concentration of 100 µg/mL against the comparatively resistant *A. hydrophila* and *A. veronii*. It is noteworthy that conducting scientific research in this area requires collaboration among materials science, microbiology, and veterinary sciences experts. The researchers can provide valuable insights into the unique properties of Mithun urine and its potential application in the production of nanoparticles. Furthermore, they can conduct appropriate analyses to evaluate the antimicrobial efficacy of the resulting nanoparticles toward bacterial pathogens in aquatic organisms. In conclusion, the suggestion of utilizing copper oxide nanoparticles derived from Mithun urine as a potential antibacterial remedy for fish bacterial infections is intriguing. However, additional research and experimentation are necessary to establish its effectiveness, safety, and feasibility.

## Data Availability

Not applicable.
